# Antitumor and immunomodulatory effects of low-dose 5-FU on hepatoma 22 tumor-bearing mice

**DOI:** 10.3892/ol.2014.1856

**Published:** 2014-02-06

**Authors:** ZHIYUN CAO, ZHIDENG ZHANG, ZHENGRONG HUANG, RONGPING WANG, AILIAN YANG, LIANMING LIAO, JIAN DU

**Affiliations:** 1 Fujian Academy of Integrative Medicine, Fujian University of Traditional Chinese Medicine, Fuzhou, Fujian 350108, P.R. China; 2 Inspection and Quarantine Technique Centre of Fujian Entry-Exit Inspection and Quarantine Bureau, Fuzhou, Fujian 350001, P.R. China; 3 Department of Integrated Chinese and Western Medicine, Fujian Provincial Cancer Hospital, Fuzhou, Fujian 350000, P.R. China; 4 Health Check Center, The Second People’s Hospital of Fujian Province, Fuzhou, Fujian 350003, P.R. China

**Keywords:** 5-FU, immunomodulatory, antitumor, mice

## Abstract

Low-dose 5-fluorouracil (5-FU), a widely used chemotherapeutic, has been reported to have immunomodulatory effects. This study aimed to evaluate the optimal dose of 5-FU that produces antitumor and immunomodulatory effects. In a hepatoma 22 tumor-bearing mouse model, 0, 10, 20 and 40 mg/kg 5-FU (i.p.) was administered for 10 days. Tumor weight and volume were measured, thymus index (TI) and spleen index (SI) were calculated, and the number of white blood cells (WBCs) and lymphocytes (LYs) were counted following treatment. The percentages of CD3^+^, CD4^+^, CD8^+^ and natural killer (NK) cells were measured by flow cytometry. In addition, the body weights of the mice were measured and the average diet consumption was calculated. Administration of 5-FU produced a potent antitumor effect in a dose-dependent manner (P<0.01). At 20 and 40 mg/kg, a significant reduction of body weight and food consumption was observed. TI and SI decreased in the 20- and 40-mg/kg groups (P<0.01) for 10 days. The number of WBCs significantly decreased in each group (P<0.01); however, the number of LYs only decreased in the 40-mg/kg group (P<0.01). Percentages of CD3^+^ and CD4^+^ cells were increased in the 10- and 20-mg/kg groups (P<0.01). Thus, 5-FU at 10 mg/kg inhibits tumor growth while maintaining the immune function of the mice. 5-FU may exert its antitumor effect at a low dose with low toxicity and stimulate the host immune system. Future clinical trials taking into account the immunostimulatory capacity of chemotherapeutic agents are desirable for certain patients.

## Introduction

Cancer patients are frequently immunologically suppressed during treatment. Chemotherapy is usually considered immunosuppressive due to its toxicity to bone marrow cells (1). However, certain chemotherapeutic agents, including leucovorin, cyclophosphamide, methotrexate and cisplatin, have been shown to activate an anticancer immune response at a low dose (2–4).

5-Fluorouracil (5-FU) is one of the most commonly used chemotherapeutics in the treatment of malignancies arising from the breast, gastrointestinal tract, head and neck (5). Over the last decades a variety of doses of 5-FU have been developed in clinical trials for the treatment of gastrointestinal tumors. These include the Mayo regimen (600 mg/m^2^ bolus) (6), FOLFOX4 regimen (400 mg/m^2^ bolus plus 600 mg/m^2^ continuous infusion) (7) and Arbeitsgemeinschaft Internistische Onkologie schedule (2,000–2,600 mg/m^2^) (8). These regimens have been shown to produce different toxicity patterns (9). For example, single-agent bolus 5-FU administered weekly, which was in the past the standard schedule and route of administration for this drug in gastrointestinal cancer, is associated with marked myelosuppression. The commonly used 5-FU/calcium folinate regimens, depending on the doses and schedules, may produce a combination of mucositis, diarrhea and myelosuppression (10). However, low-dose (250–300 mg/m^2^/day) continuous infusion of 5-FU is associated with little myelosuppression (11). In general, the incidence of grade 3–4 toxicity (mainly neutropenia, diarrhea, mucositis and hand-foot syndrome) increases with higher systemic exposure to 5-FU (12).

However, several studies have shown that patients receiving low-dose 5-FU have favorable survival rates without toxicity (13,14). These studies suggest that the favorable outcome in patients receiving low-dose 5-FU may be due to its immunomodulatory effects and enhancement of the immunologic function of the host. However, whether 5-FU exerts its antitumor action at a low dose that enhances the host’s immune function, as well as having minimal side effects, has not been reported.

In this study, we evaluated whether there was an optimal dose of 5-FU with antitumor and immunomodulatory effects, without evident side effects in hepatoma 22 (H22) tumor-bearing mice.

## Materials and methods

### Reagents

5-FU (25 mg/ml) was purchased from Shanghai Amino Acids Company (Shanghai, China) and diluted to 10 mg/ml with normal saline (NS). Peridinin chlorophyll protein complex-conjugated hamster anti-mouse CD3, fluorescein isothiocyanate-conjugated rat anti-mouse CD4, phycoerythrin (PE)-conjugated rat anti-mouse CD8 and PE-conjugated rat anti-mouse CD49 were purchased from BD Biosciences (San Jose, CA, USA).

### Mice

Male Institute of Cancer Research mice (body weight, 18–22 g) were purchased from the School of Basic Medical Science, Peking University (Beijing, China). The animals were maintained in a pathogen-free facility (23±2°C, 55±5% humidity). All procedures on treating mice were performed according to the law on Animal Care Guidelines, and the Animal Care Committee of Fujian University of Traditional Chinese Medicine (Fuzhou, China) approved the study protocols. The murine H22 cell line was from the School of Basic Medical Science, Peking University. Cells were cultured in Dulbecco’s modified Eagle’s medium (Gibco Laboratories, Grand Island, NY, USA) supplemented with 10% fetal bovine serum (Gibco Laboratories), penicillin and streptomycin (10,000 U/ml; Gibco Laboratories) in a humidified atmosphere with 5% CO_2_ at 37°C.

### Tumor xenograft

The hepatoma model was established by subcutaneous inoculation of H22 cells (1×10^6^ cells per mouse) into the right flank of mice. Then, 48 h after inoculation, mice were randomly divided into four groups (n=6) as follows: Control group (NS, i.p.), 10-mg/kg group (i.p.), 20-mg/kg group (i.p.) and 40-mg/kg group (i.p.). On day 10, peripheral blood was collected from the orbital plexus and tumors were excised. The tumor volume (TV) was calculated according to the following formula: $\text{TV }({\text{mm}}^{3})={\text{d}}^{2}\times \text{D}/2$, where d and D were the shortest and longest diameters, respectively.

### Food intake and body weight

Each cage of mice was provided with daily food. The following day, the remaining food was collected and weighed. The daily food intake was calculated by subtracting this value from the amount of diet provided the previous day. The well-being of the mice was monitored daily and the body weight was measured at baseline and after 10 days of treatment.

### Biochemical assay

A capillary pipette containing anticoagulant [ethylene diamine tetraacetic acid (EDTA) for cell counting and heparin for flow cytometry] was inserted in the lateral canthus and blood was collected from the retroorbital sinus. A 20-μl whole blood sample was collected and blood cells were counted using an automated Blood Cell Counter (Abbott Laboratories, Abbott Park, IL, USA).

### Thymus index (TI) and spleen index (SI)

The thymus and spleen were collected from the mice, washed with phosphate-buffered saline (PBS) and weighed. TI and SI were calculated according to the following formulae: TI=thymus weight (mg)/body weight (g)×100; SI=spleen weight (mg)/body weight (g)×100.

### Assay for percentage of immune cells

Heparin-coated blood (100 μl) was added to a tube and incubated with fluorochrome-conjugated antibodies, respectively, in the dark for 10 min. The 2.5 μl antibodies used included a combination of CD3/CD4, CD3/CD8 and CD3/CD49. Erythrocytes were lysed by red blood cell lysis buffer (0.155 mol/l ammonium chloride, 0.01 mol/l potassium bicarbonate, 0.1 mmol/l EDTA and 1% paraformaldehyde in PBS) for 10 min. After washing with PBS, the samples were resuspended with 500 μl PBS and analyzed using a FACSCalibur™ flow cytometer with CellQuest™ software (BD Biosciences).

### Statistical analyses

Statistical analysis was performed using SPSS 16.0 (SPSS, Inc., Chicago, IL, USA). Data are presented as the mean ± standard deviation. One-way analysis of variance was used and significant differences were analyzed by Dunnett’s multiple comparison test to compare with the control group. P<0.05 was considered to indicate a statistically significant difference.

## Results

### Effects of 5-FU on tumor growth in H22-bearing mice

**Figure 1:**
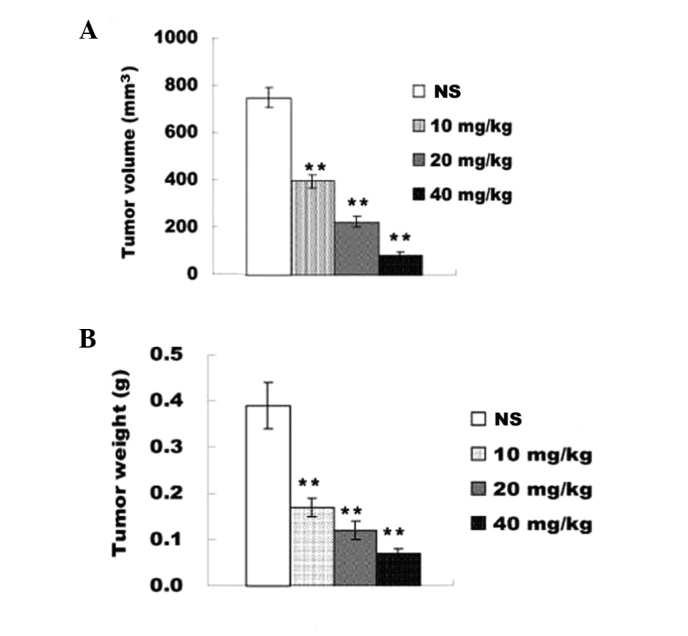
Effects of different doses of 5-FU on H22 solid tumor growth. H22 tumor-bearing mice were treated with NS (control group) or 5-FU (10, 20 and 40 mg/kg). The antitumor effect of each regimen was evaluated by measuring (A) tumor volume and (B) tumor weight. The results presented are the mean ± standard deviation (n=6). ^**^P<0.01, compared with the NS group. 5-FU, 5-fluorouracil; NS, normal saline.

Tumor-bearing mice were randomly divided into four groups as described in Materials and methods and treated with 5-FU for 10 days. After 10 days, tumor weights were 0.39±0.05, 0.17±0.02, 0.12±0.02 and 0.07±0.01 g in the control, 10-, 20- and 40-mg/kg groups, respectively, which exhibited a reduction by 56 (P<0.01), 69 (P<0.01) and 82% (P<0.01), respectively. TV demonstrated the same tendency as tumor weight among the four groups (Fig. 1).

### Food intake and body weight

**Figure 2:**
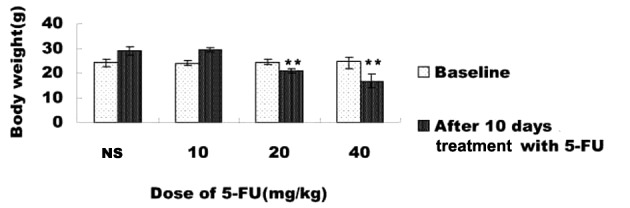
Effects of different doses of 5-FU on body weight. H22 tumor-bearing mice were established and treated for 10 days with different doses of 5-FU. The body weight gain was calculated after treatment for 10 days. The results presented are the mean ± standard deviation (n=6). ^**^P<0.01, compared with the NS group. 5-FU, 5-fluorouracil; NS, normal saline.

**Figure 3:**
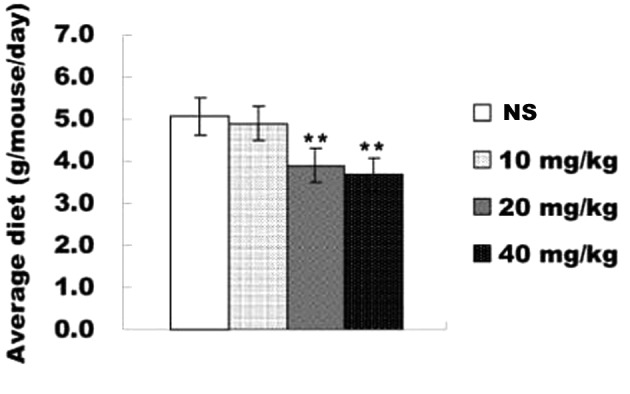
Effects of different doses of 5-FU on average diet. H22 tumor-bearing mice were established and treated for 10 days with different doses of 5-FU. The diet weight was measured and the average consumption per mouse is shown. The results presented are the mean ± standard deviation (n=6). ^**^P<0.01, compared with the NS group. 5-FU, 5-fluorouracil; NS normal saline.

We monitored the body weight at baseline and after treatment, as well as diet consumption every day, which are widely used to assess gross toxicity of a test compound. There was no statistically significant difference in body weight among the groups at baseline. Following treatment with 5-FU for 10 days, the body weight was similar between the control group and the 10-mg/kg group. However, body weight was significantly lower in the 20- and 40-mg/kg groups compared with the control group (P<0.01; Fig. 2). The average diet consumption of the control and 10-mg/kg groups were similar; however, it was smaller in the 20- and 40-mg/kg groups (P<0.01 vs. control group; Fig. 3).

### Effects of 5-FU on the number of WBCs

To investigate the role of 5-FU on the distribution of circulating WBCs, whole blood WBC and LY levels were counted at day 10. As shown in Table I, WBC number was decreased significantly in the 10- (P<0.01), 20- (P<0.01) and 40-mg/kg (P<0.01) groups compared with the control group. LY number was only decreased in the 40-mg/kg group (P<0.01).

### Effects of 5-FU on percentage of LYs in peripheral blood

To evaluate the effects of 5-FU on host immune system, percentages of LYs [CD3^+^, CD4^+^, CD8^+^, natural killer (NK) and B cells] were measured after mice were treated with different doses of 5-FU for 10 days. As shown in Table II, the percentages of CD3^+^ and CD4^+^ cells were higher in the 10- (P<0.01) and 20-mg/kg (P<0.01) groups compared with the control group. Mice in the 40 mg/kg group had the lowest percentages of CD3^+^ and CD4^+^ cells (P<0.05 vs. control group). The percentage of CD8^+^ cells was only decreased in the 40-mg/kg group (P<0.01). However, the number of NK cells was decreased in the 10- (P<0.01) and 20-mg/kg (P<0.05) groups compared with the control group.

### Effects of 5-FU on TI and SI in H22-bearing mice

**Figure 4:**
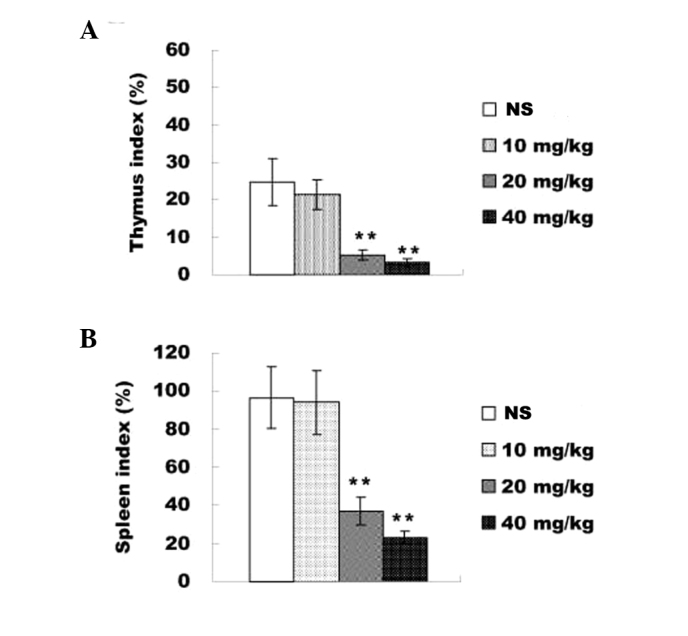
Effects of different doses of 5-FU on TI and SI. The thymus and spleen were washed with phosphate-buffered saline and weighed. (A) TI was calculated according to the following formula: TI=[thymus weight (mg)/body weight (g)]×100. (B) SI was calculated according to the following formula: SI=[spleen weight (mg)/body weight (g)]×100. The results presented are the mean ± standard deviation (n=6). ^**^P<0.01, compared with the NS group. 5-FU, 5-fluorouracil; TI, thymus index; SI, spleen index.

We also examined the effect of 5-FU on TI and SI in H22 tumor-bearing mice. After 10 days, TI and SI decreased in the 20- (P<0.01) and 40-mg/kg (P<0.01) groups compared with the control group (Fig. 4).

## Discussion

The adverse effects of 5-FU include severe immunosuppression due to inhibition of hemotopoietic cell proliferation (13). A previous study demonstrated that low-dose chemotherapeutic drugs have a positive effect on various solid tumors. Kobayashi *et al* reported that low dose leucovorin plus 5-FU treated seven colorectal cancer patients for a long duration without toxicity (14). The authors further reported that low dose leucovorin plus 5-FU may improve host immunity in certain patients (15).

Our results showed that 20 and 40 mg/kg 5-FU induced a significant loss of body weight and reduction in diet consumption. Codacci-Pisanelli *et al* reported that 15 and 20 mg/kg caused ~25% loss of weight in mice (16). Vichaya *et al* also reported that injection with 20 and 40 mg/kg 5-FU every other day in C57BL/6J mice resulted in weight loss and suppressed food consumption (17). Our results are consistent with these studies.

Certain chemotherapeutics have been reported to have benefit for patients at a low dose, including cyclophosphamide (CY), methotrexate (MTX) and cisplatin (Cis). Daily oral administration of low dose CY (50 or 100 mg/day) is effective in patients with advanced solid tumors, since CY is capable of not only selectively ablating circulating T regulatory (Treg) cells, but also recovering the function of conventional T and NK cells. T and Treg cells coregulate the activity of each other, which leads to the restoration of peripheral T-cell proliferation and innate killing activities (2,18–20). Shen *et al* demonstrated that low-dose, metronomic chemotherapy with Cis (0.6 mg/kg/day) dramatically inhibits tumor growth without apparent body weight loss and significant upregulation of Fas (CD95) mRNA and protein in SW480 colon cancer cells and oral cancer cell lines. It is possible for low-dose Cis to attenuate inflammatory responses by inducing Fas expression on effector T cells (21–23). We found that although 5-FU at 10 mg/kg demonstrated moderate antitumor effects, no severe side effects were observed. In addition, 10 mg/kg 5-FU may enhance host immune function as indicated by increased percentages of CD3^+^ and CD4^+^ cells. A dose of 10 mg/kg for tumor-bearing mice is approximately equal to 0.83 mg/kg for cancer patients. In clinical settings, 5-FU is usually administered at 12 mg/kg/day for 4 days (max, 800 mg/day) followed by 6 mg/kg/day every other day for 4 days. Kobayashi *et al* reported improved immune function in cancer patients receiving low-dose 5-FU (14,15).

In conclusion, our study demonstrated that improved immune function achieved by low dose 5-FU may translate into an antitumor effect. Future clinical trials are required to confirm this finding.

## Figures and Tables

**Table I:** Number of WBCs and LYs in peripheral blood of H22-bearing mice.

Dose (mg/kg)	WBC (×10^9^/l)	LY (×10^9^/l)
NS	8.0±1.2	3.5±1.1
10	4.6±0.5^a^	3.6±0.3
20	4.4±0.4^a^	3.5±0.4
40	1.1±0.4^a^	0.9±0.4^a^

Dose was administered over a 10-day period. Results are presented as the mean ± standard deviation, n-=6.

a P<0.01, compared with the NS group.

NS, normal saline; WBC, white blood cell, LY, lymphocyte.

**Table II:** Percentages of CD3^+^, CD4^+^, CD8^+^ and NK cells in peripheral blood of H22-bearing mice.

Dose (mg/kg)	CD3^+^ (%)	CD4^+^ (%)	CD8^+^ (%)	NK (%)
NS	48.5±3.5	36.4±3.9	12.1±1.6	14.0±3.3
10	82.7±1.8^a^	71.7±0.4^a^	11.0±1.3	6.4±2.9^a^
20	81.0±8.5^a^	71.3±8.2^a^	9.7±6.9	9.9±11.2^b^
40	38.8±6.6^a^	34.4±7.4	4.4±1.8^a^	12.0±3.7

Dose was administered over a 10-day period. Results are presented as the mean ± standard deviation (n=6).

a P<0.01 and

b P<0.05, compared with the NS group.

NK, natural killer; NS, normal saline.

## Abbreviations

5-FU: 5-fluorouracil
H22: hepatoma 22
WBC: white blood cell
LY: lymphocyte
TI: thymus index
SI: spleen index
TV: tumor volume
PBS: phosphate-buffered saline
